# A Contribution to the Study of Deafness

**Published:** 1901-05

**Authors:** Ross P. Cox

**Affiliations:** Aurist and Oculist to the Georgia School for the Deaf


ATLANTA

### Journal-Record of Medicine.

Successor to Atlanta Medical and Surgical Journal, Established 1855, and Southern Medical Record, Established 1870.

VOL. III. MAY, 1901. No. 2.

BERNARD WOLFF, M.D., EDITOR, Nos. 319–20 Prudential.

Published Monthly.

M. B. HUTCHINS, M.D., BUSINESS MANAGER, No. 64 Marietta St.

### ORIGINAL COMMUNICATIONS.

### A CONTRIBUTION TO THE STUDY OF DEAFNESS.*

BY ROSS P. COX, A.B., M.D.,

Aurist and Oculist to the Georgia School for the Deaf.

I beg to submit to your consideration some of the results of my recent work as aurist and oculist to the Georgia School for the Deaf.

Except as elsewhere stated, the time has been devoted to the preliminary work of examinations of the ears, noses, throats, eyes and accessory parts of the pupils, and to the making of permanent records and suggestions for treatment on forms prepared for this purpose and bound into a book.

I have found the performance of this task an exceedingly time-consuming and patience-exacting process, requiring from two to four times as much time as would be necessary with those of fair speech and hearing. These facts account for the apparently slow progress.

*Read before the Medical Association of Georgia, at Augusta, April, 1901.

I have examined the ears and related organs of 68 pupils.

Of these 39 are girls and 29 boys. Their average age is thirteen and one-half years.

There was one case of suppurating mastoiditis, with fistulous discharge and sharp inflammation of the adjacent lymphatics.

This patient improved so much under mild measures before permission to operate could be secured that surgery was not used. The drum of the ear concerned was perfectly normal.

There was stenosis of the external canal of one ear.

The external canals of seven ears were filled with hard wax, which was removed in the usual way.

The following diseases have been given by the parents or guardians as the causes of the deafness of these 68 children, viz.: measles, 2; catarrh, 3; whooping-cough, 1; illness in infancy, 1; falls in infancy, 2; quinine, 1; rising in the head, 5; scrofula, 1; meningitis, 4; fever, 1; grippe, 1; born deaf, 41; and from causes unknown, 5.

Based upon all the information, both subjective and objective, I have been able to obtain, I have in a general way classified the causes of deafness pathologically as follows, viz.: Those associated with non-suppurative inflammation of the middle ear, with adenoid vegetations of the nasopharynx, 15, and without adenoids, 6; those originating in the suppurative inflammation of the middle ear, with adenoids, 7; without adenoids, 2; those due to congenital lesions of the inner ear, the nerve tracts or brain centers, 24; perception lesions due to meningitis, 3; perception lesions, not congenital, and not originating in the conduction apparatus, but from causes undermined, 11.

It will be noted that the 30 cases of middle ear inflammation were associated with adenoids in 22 instances, which constitutes 33 per cent. of the total number of cases. Attention is also called to the fact that the pathological classification gives the number of the congenital deaf as 24, in contrast to that of the parents and guardians, which places the born deaf at the much higher figure of 41.

My investigations encourage the opinion that even when the child had deaf parents or deaf brothers and sisters, the cause of deafness is not always due to prenatal defects of the ear or brain, but not infrequently to an inherited tendency to disease, as, for instance, that of lymphoid hypertrophy, resulting in adenoids, whose important rôle as a cause of deafness is now so definitely established. Some of these children with deaf parents or a deaf brother or sister, according to the testimony of the parents, did not become deaf before a certain age.

For example, I have records of five pupils, representing five different families, who have blood-related parents and other deaf relatives, yet, who did not become deaf before the ages of two years, thirteen months, nine months, one year, and time unknown, respectively, and whose parents give as causes of deafness high fever, catarrh, rising in the head and causes unknown. I have also notes of two other unrelated pupils, who have deaf brothers or sisters, who became deaf at the ages of eighteen months and one year, and the causes given are rising in the head and unknown.

Of these 7 children, 6 show evidence of severe and one of moderate prolonged middle ear disease, 6 have adenoids, 4 have nasal stenosis and 4 have enlarged faucial tonsils.

Consanguineous marriages are well known to often produce a scrofulous or tubercular tendency and predisposition to lymphoid development, resulting often in large adenoids and tonsils, which may produce deafness at so early an age that the inability to hear was supposed to have been congenital.

It is highly probable that deafness, like many other defects of unsound or consanguineous parentage, is often not in fact congenital, but a result of lessened vitality, impaired physiological energy, which allows the parts concerned to fall an early and easy prey to untoward influence.

Eight children of the 68 have parents who are first cousins, two third and one fourth cousins, representing in all ten families.

There are 16 pupils who have deaf relatives.

Twenty-three pupils out of the 68 had one or more practically normal ear-drums, making 38 absolutely or nearly normal drums to 98 diseased ones.

The cause of deafness of the 23 pupils, with one or more normal drums, as given by the parents, illness in infancy 1, rising in head

1, meningitis 2, fall 1, quinine 1, catarrh 2, congenital 13, and unknown 2.

Ninety-eight drums showed more or less decided effects of disease, such as great retraction, sinking, hypertrophy, atrophy, scars, chalk deposits, perforations, more or less fixation of ossicles, etc.

In only two cases was there existing suppuration of the middle ear, the pathological process having, for the most part, expended itself and become more or less extinct.

I have felt justified in placing the number of children who have adenoid vegetations at 42, though in only about 25 were the growths actually seen or felt, the others being inferred from follicular pharyngitis, mouth-breathing, snoring, etc.

Thirty pupils had enlarged tonsils. Twenty-six had nasal stenosis on one or both sides, due to deviated septa, spurs, enlarged turbinates, or general narrowing.

Of the 41 cases given by parents as congenital, 29 had bad drums and 19 had adenoids. Only 4 of the 12 with normal drums had adenoids.

The hearing tests gave important and interesting results. These were made with the human voice, Galton's whistle, and a series of 2 tuning forks, C-2, C, and C-3.

In only the last 37 cases did I keep records of the patients' statement of the hearing. This gave 37 as totally deaf, and 11 as partially so. My examination of the 37 so-called totally deaf, by the fork test, showed only two to be absolutely deaf, 11 very nearly so, and 24 with fair or good hearing for some or all of the forks.

If we take the 65 cases in which fairly satisfactory tests with forks were made, we may state that 6 heard all the forks in both ears, 5 heard all in one ear, 8 were deaf to all forks in one ear, and about 10 practically heard no forks in either ear.

The C-2 fork was heard by air only in two of the 130 ears, by bone only in 43 ears, and by both air and bone in 47 ears.

The C fork was heard by air only in 28 ears, by bone only in 1 ear, and by both air and bone conduction in 60 ears.

The C-3 fork was heard by air only 17 times, by bone only 5 times, and by both bone and air 28 times.

In 32 of 68 cases the right ear was the better hearing ear, and in 16 the left ear heard best.

In 106 ears the loud voice directly in the ear was absolutely inaudible in 10 ears, it was heard only as a noise in 72 ears, vowels were heard in 14 ears, words in 7 ears, and sentences in 3 ears.

In 136 ears the Galton whistle was heard at some point in 72.

The lowest fork was heard better by air conduction in only 5 cases out of 136, while by bone conduction the lowest fork was heard best in 94 cases.

The highest fork was heard best by air in 29 cases, and, by bone, in 17 cases.

Some cases had perception of the loud voice, but no perception of the forks.

There is good reason for believing that some of those who apparently failed to perceive any sound could, by other sounds or a little drilling, be aroused to some degree of hearing. My belief is that the per cent. of children examined who are absolutely deaf to any and all perception of sound is little over and probably under 5. This fact is of great practical importance.

During the last 20 years the method of teaching the deaf has been largely revolutionized. Experience has established that spoken language and lip-reading do most to minimize the natural disadvantages of the deaf. In all promising cases the present rule is to accomplish this slowly, laboriously, artificially, mechanically, without any use of, or dependence upon, any possible remnant of hearing, solely through the sight and touch.

During recent years the representatives of yet another system, not new but greatly improved and revised, have brought the auricular method more and more into favorable notice. The simple plan of this system is to arrive at speech by the natural route of any possible remnants of hearing. It proposes to make practical use of any manifest hearing and to arouse the latest perception of the psychically deaf by a system of methodical hearing-exercise. In the general wreck of the hearing precious fragments of the same are often overlooked, covered-up and lost, like treasures of art under heaps of earth and rubbish. The auricular method seeks to exhume these buried treasures and restore them to their appointed places in the temples of the mind.

Experts estimate that 20 to 80 per cent. of the inmates of institutions for the deaf could be benefited by this system of improving possible hearing by exercising it. Certain it is that many persons, hitherto supposed to be totally deaf, have by this system been brought not only to speak, but actually hear words and sentences, and thus have their usefulness and happiness vastly increased.

The most distinguished and successful representative of this method at present is Prof. Urbantschitsch of Vienna, whose pupil I had the good fortune for a time to be, and by whose great and genuine success I was profoundly impressed. Betzold of Munich, Currier of New York, Gillespie of Omaha, and many others have achieved signal success along this line and are using it with enthusiasm and great satisfaction. Not only do these pupils acquire readier and more certain interchange of ideas with hearing people, but also modulation, pleasanter voices, and the protection from accidents that even very imperfect hearing gives. The drawbacks to the method are the untiring patience, the high skill, and the great time that its proper use requires.

The principles of this method are practically for all degrees of impaired hearing, and a knowledge of them is important for all physicians, as the probable advisers of affected persons.

But whether we teach speech naturally through the ear, or artificially through sight and touch, certain it is that stopped noses, adenoids of the nasopharynx, enlarged or inflamed faucial tonsils, and all other agencies that block or fill the resonating spaces, clog and embarrass the tongue and palate and promote disease of the whole body, but especially of the organs of speech, respiration and hearing, must be gotten rid of if we hope to approximate the best results.

It is equally certain, if experience teaches anything, that timely and proper management of many of these conditions is now practicable, obligatory, and sure to prevent much deafness; for I am convinced that if deafness is to be decreased as blindness has been, it will be done in the same way, far more by prevention rather than cure. Of course I speak of confirmed deafness, such as is found in institutions for the deaf, where, I think, only a relatively small per cent. can receive any benefit to the hearing by any treatment.

Since the deaf depend so largely upon vision, the condition of their eyes is of special importance.

I have partially or completely examined the eyes of 68 children, and this number probably includes about all the noteworthy defects to be found among the 130 white pupils, as all who, by their own statement or by the observation of the teachers, were supposed to have any eye trouble are here included.

Of the 68, 8 had distant vision above normal, 36 were normal, 2 had $\frac{1}{3}$ vision, 2 had $\frac{1}{2}$ vision, 11 had $\frac{2}{3}$, 2 had 2-5, 1 had 1-10, 2 had 1-30, 1 was blind in one eye and had $\frac{2}{3}$ in the other, 1 had $\frac{2}{3}$ in one eye and 1-5 in the other, 1 had $\frac{1}{2}$ in one eye and 1-10 in the other, and one had $\frac{1}{3}$ in one eye and 1-20 in the other.

Forty-five, for bad vision or clear symptoms of eye-strain, require refraction or other treatment. Seven have been fully refracted. Of these 6 required glasses, and the seventh was so nearly blind from chorioretinitis that he was taken from school for rest and treatment.

There are 15 or 20 other pupils whose need for attention to eyes is urgent.

I find that the refraction of these deaf children requires many times the time, patience and care necessary for hearing children. One is forced to rely largely upon objective methods. I was greatly assisted by the shadow test and my ophthalmometer, which I brought down to the institution.

